# Monetary rewards modulate inhibitory control

**DOI:** 10.3389/fnhum.2014.00257

**Published:** 2014-05-12

**Authors:** Paula M. Herrera, Mario Speranza, Adam Hampshire, Tristán A. Bekinschtein

**Affiliations:** ^1^Laboratoire ECIPSY - EA 4047, Université de Versailles Saint Quentin en YvelinesVersailles, France; ^2^Grupo de Investigación en Neurociencias (NeURos), Facultad de Ciencias de la Salud, Universidad del RosarioBogotá, Colombia; ^3^Grupo de Investiga, Laboratorio de Psicología Experimental, Facultad de Psicología, Universidad El BosqueBogotá, Colombia; ^4^Child and Adolescent Psychiatry Department, Centre Hospitalier de VersaillesVersailles, France; ^5^Division of Brain Sciences, Department of Medicine, Imperial College LondonLondon, UK; ^6^Medical Research Council-Cognition and Brain Sciences UnitCambridge, UK

**Keywords:** reward, inhibition (psychology), cognitive control, stop signal task, behavioral analysis

## Abstract

The ability to override a dominant response, often referred to as behavioral inhibition, is considered a key element of executive cognition. Poor behavioral inhibition is a defining characteristic of several neurological and psychiatric populations. Recently, there has been increasing interest in the motivational dimension of behavioral inhibition, with some experiments incorporating emotional contingencies in classical inhibitory paradigms such as the Go/NoGo and Stop Signal Tasks (SSTs). Several studies have reported a positive modulatory effect of reward on performance in pathological conditions such as substance abuse, pathological gambling, and Attention Deficit Hyperactive Disorder (ADHD). However, experiments that directly investigate the modulatory effects of reward magnitudes on the performance of inhibitory tasks are scarce and little is known about the finer grained relationship between motivation and inhibitory control. Here we probed the effect of reward magnitude and context on behavioral inhibition with three modified versions of the widely used SST. The pilot study compared inhibition performance during six blocks alternating neutral feedback, low, medium, and high monetary rewards. Study One compared increasing vs. decreasing rewards, with low, high rewards, and neutral feedback; whilst Study Two compared low and high reward magnitudes alone also in an increasing and decreasing reward design. The reward magnitude effect was not demonstrated in the pilot study, probably due to a learning effect induced by practice in this lengthy task. The reward effect *per se* was weak but the context (order of reward) was clearly suggested in Study One, and was particularly strongly confirmed in study two. In addition, these findings revealed a “kick start effect” over global performance measures. Specifically, there was a long lasting improvement in performance throughout the task when participants received the highest reward magnitudes at the beginning of the protocol. These results demonstrate a dynamical behavioral inhibition capacity in humans, as illustrated by the reward magnitude modulation and initial reward history effects.

## Introduction

Behavioral inhibition is an essential component of goal-oriented behavior, allowing the suppression of a pre-potent behavior in order to switch to a more suitable action when conditions change. Cancelling a planned action is also called “executive inhibition” (Nigg, 2001), as part of the inhibitory control network.

Measuring inhibition under experimental conditions has evolved progressively on the basis of core concepts as the negative priming (Tipper, 2001), interference control (Salo et al., 2001), mental withholding (Brass and Haggard, 2007), and allocation of attention (Hasher et al., 1999) among others. A non-exhaustive list of inhibition classical tests includes the Flankers (Wendt et al., 2014), the Go/NoGo (Bokura et al., 2001), the Continuous Performance Task (Ridderinkhof et al., 2004), and the Stop Signal Task (SST; Aron et al., 2003). Each of these tasks highlights a particular aspect of the inhibitory processes (for a review in the inhibition tasks, see MacLeod, 2007).

The SST presents a frequent Go stimulus (left or right), and a less frequent Stop stimulus between Go trials. It is widely considered that the main executive process of this task is the cancellation of the on-going action being triggered by a Go stimuli (Logan, 1994).

The SST is one of the most widespread measures of inhibitory control in the cognitive sciences (Li et al., 2006; Alderson et al., 2008; Chikazoe et al., 2009). It has long been used to investigate cognition in healthy individuals (Ramautar et al., 2004; Clark et al., 2005; Lansbergen et al., 2007; van Gaal et al., 2009), and is used as a diagnostic tool in several pathological conditions including Attention-Deficit/Hyperactivity Disorder (ADHD) (Stevens et al., 2002; Nichols and Waschbusch, 2004; Sonuga-Barke, 2005), Conduct Disorder (CD) (Oosterlaan et al., 1998), Oppositional Defiant Disorder (ODD) (Albrecht et al., 2005), substance abuse (Smith and Mattick, 2013), and personality disorders (Lipszyc and Schachar, 2010).

The SST is designed to enable the measurement of the inhibition process through its gold standard measure: the Stop Signal Reaction Time (SSRT). This score measures the time required for an individual to successfully stop their initiated action. Specifically successful behavior inhibition leaves no report (it is the lack of a response), and hence the inhibition measure has to be estimated by other behavioral markers closely related and dependent on the inhibition process. The SSRT is therefore calculated as the probability of inhibition (PI) (Liotti et al., 2005; Schmajuk et al., 2006) or the subtraction of the Mean reaction time (MRT) minus the Stop signal delay (SSD) (formulae SSRT = MRT − SSD) (Kok et al., 2004). The SSRT score is given in milliseconds, reflecting the time from the presentation of Go signal at which one starts to fail. In other words: how late can you receive the order to stop the ongoing action.

Both the MRT and the SSD are direct measures allowing an indirect calculation of the behavioral inhibition performance. Beyond their use on the SSRT calculation, these measures can also give crucial hints about the behavioral adjustments through the inhibition task (van Boxtel et al., 2001; Band et al., 2003). The relevance of these two measures will be discussed later in detail.

Besides these time related measures, the SST provides other useful information about the inhibition profile as the accuracy and number of performance errors (failed Go's, failed Stop's, left-right precision errors).

The SSRT—provides a sensitive behavioral marker that can be used to compare control groups with impulsivity disorders (Lijffijt et al., 2004). It has been shown that the ability to inhibit a response is present from early ages and that the SSRT improves through development (Carver and Scheier, 2001) until becoming a stable and individual measure in adult healthy participants (Cohen and Poldrack, 2008). SSRT values around 200 ms have been described as the normal range for adults (Logan and Cowan, 1984). SSRT beyond 400 ms have been reported in young children, elderly, and impulsive participants, as well as hyperactive children (Winstanley et al., 2006). SSRT can vary according to frontal lesions (Aron et al., 2003), and can be consistently altered in disorders such as pathological gambling (Lawrence et al., 2009), psychopathic personality (Masui and Nomura, 2011), or Attention Deficit/Hyperactive Disorder (ADHD) (Oosterlaan and Sergeant, 1998; Stevens et al., 2002; Banaschewski et al., 2003).

Previous works have stated that behavioral inhibition performance reaches mature development after childhood and it has been suggested that a similar behavioral trait should be evident across a range of contexts (Williams et al., 1999; Rubia et al., 2007). Nevertheless, some experimental results suggest that inhibition can change in response to emotional states due to feedback contingencies (Bechara et al., 1994), fear (Bush et al., 2000), or other motivational influences (Pessoa et al., 2012).

Motivation refers to the volitional engagement in a task and can come from either an internal and/or an external source (Panksepp, 2003). Character and temperament theories propose a distinction among individuals with an accentuated need for external rewards, while others would exhibit a more internal motivation driven behavior (Derryberry and Rothbart, 1997). Even if it is assumed that everyone has a distinctive behavioral pattern, specific situations can lead to unusual reactions, leading to popular phrases such as “money talks” (Living Colour, 1991) or “everybody has a price” (Jessie, 2011). The individual need of external incentives is part of the basic-stimulus response mechanism known as “reward dependency” (Cloninger, 1987), and can be overexpressed in pathological conditions such as gambling and compulsive buying (Avila and Parcet, 2001). Moreover, both clinical and experimental evidence support the view that ADHD children are particularly affected by immediate and salient rewards when engaging with a task (Michel et al., 2005; Sonuga-Barke, 2005; Potts et al., 2006; Groom et al., 2010; Luman et al., 2010).

The influence of motivation over behavior inhibition may be achieved through different strategies. One of the most prevailing is the “aversive/approach” system, allusive to the “Behavioral Inhibition System/Behavioral Activation System” (BIS/BAS) model (Gray, 1987; Quay, 1993). For instance, increased response times are observed when comparing neutral against punishment contingencies. This strategy can be convenient to heighten the likelihood of successful inhibition when avoiding punishment. In the case of reward contingencies, behavioral inhibition can be adjusted to increase the sum of fruitful inhibitions, thereby increasing the number of rewards (Boehler et al., 2012).

A range of prefrontal, sub cortical, and limbic structures have been implicated in behavioral inhibition in different task contexts. For example, premotor areas (Peterson et al., 1999), basal ganglia (Brown et al., 1999), and Anterior Cingulate Cortex (ACC) (Braver et al., 2001) are strong candidates as core anatomical structures enabling inhibitory motor control during the SST and its various analogs. By contrast, activation of the orbitofrontal cortex (OFC), caudate nucleus (Elliott et al., 2000), and limbic system structures (Etkin et al., 2006) has been reported when behavioral inhibition involves “hot” or emotional choices in response to punishment or reward. It has been suggested that the dorsolateral prefrontal (DLPF) cortex is involved in planned or “proactive” inhibition (Bechara et al., 1994; Dias et al., 1997). Executive and motivational inhibitory circuits are interconnected and share some anatomical pathways, but they also rely on independent structures (Nigg, 2000).

Despite being the focus of much research, behavioral inhibition and the mechanisms by which it is modulated remain poorly understood. Indeed, many authors have highlighted the inherent difficulty in taking a specific measure of inhibition due to other simultaneous processes that are tapped by classical inhibitory paradigms including perception, attention, and response planning (Rubia et al., 2003; Chen et al., 2013). Positive or negative emotions may interfere with the inhibition processes (Kalanthroff et al., 2013), as well as other elaborate cognitive processes related to education, culture, and environmental factors (Immordino-Yang and Damasio, 2007). Emotions may influence Inhibition by high order cognitive process such as reasoning, labeling, and voluntary modulation (van Reekum and Schaefer, 2011).

Even though the recent literature recognizes the role played by motivational aspects over inhibitory processes, few studies have explored the links between emotion and the inhibitory components of executive control and, more specifically, the effect of reward magnitude and context on inhibition capacity (Kalanthroff et al., 2013). The majority of those reports state simple contrasts, where inhibition is analyzed under rewarded vs. no-rewarded contingencies. Others have compared punishment against reward (Rubia et al., 2005), contrasting emotions such as erotic or painful stimuli (Yu et al., 2012), fear (Verbruggen and De Houwer, 2007; Sagaspe et al., 2011), or goal conflict (Neo et al., 2011).

The recent theoretical and experimental literature proposes the existence of two independent pathways for behavioral inhibition. The “cool” pathway, corresponding to deliberate executive control, and the “hot” pathway, related to affective or motivational modulations (Nigg, 2001; Zelazo et al., 2003). It has been suggested that most of the inhibitory tasks involve both executive and motivational pathways albeit to widely varying degrees (Geurts et al., 2006).

The overarching goal of this study was to obtain a clearer understanding of how reward and reward context modulate behavioral inhibition performance during the SST. The longer-term goal is to reproduce these studies with neuroimaging and EEG in order to explore the neural underpinnings of these effects. More specifically, we explored the motivational modulation of behavioral inhibition in normal adults using a SST with reward. A close temporal manipulation of reward size and contingencies was used to obtain a better understanding of the motivational dynamical adjustments of behavioral inhibition capacities. We highlight two specific aims: to clarify how important the magnitude of reward is (no reward, low or high reward) and what is the nature of that relationship. Is the value of the reward itself, strong enough to induce a similar level of behavioral inhibition performance, that is, a trait independent of context? The second aim was to determine whether the history of presentation of different reward magnitudes modulated behavior. What happens when opposing contrasted reward magnitudes at different times? Is there any difference when receiving a given reward at the beginning or at the end of the task?

On the basis of previous studies using reward contingencies in inhibition tasks in adults (Boksem et al., 2008; De Pascalis et al., 2010; Pessoa et al., 2012; Yu et al., 2012) one would hypothesize that the presence of a reward should improve inhibition performances compared to a neutral feedback. Hence, we expect to find that higher rewards would have a higher impact on inhibition independently from the order of presentation. Moreover, we predicted a reciprocal modulation effect relying on the history of presentation of rewards: a straight improvement in performances when presenting progressively increasing rewards, and a disengaging effect when moving from high to low reward.

## Methods

### Participants

Young adult participants were recruited by informal community announcements among the staff and medicine school students attending at the Versailles General Hospital, the undergraduate students of the University of Nantes, and a mailing list of volunteers from the MRC-CBU in Cambridge.

One hundred and one participants were recruited (21 for pilot study, 41 for study one, and 39 for study two). The combined mean age for both men and women participating from the study was 24.7 (age range 20–33, *SD* = 4.5; sex ratio = 1.1), and had at least 2 years of Higher Education. They were screened for past and current psychiatric disorders ADHD, depression or bipolar disorders and schizophrenia, as these were part of the exclusion criteria. All participants gave written consent according to the procedures of the Ethical committee of the Versailles General Hospital (France) and the Cambridge Research and Ethics Committee (UK).

Before statistical analysis, all time responses (MRT, SSD, and SSRT) were screened for extreme values. A cutting point of ±2 standard deviations from the mean response value was considered as outlier. Three participants were excluded from study one following this criterion.

### Procedure

Participants performed the experiment in a quiet room with a desk and a computer. After a short clinical interview to verify medical history, they were given a folder with questionnaires to fill up, written information about the study and a consent form in paper form.

All participants underwent a single 8 min acquisition block of a Go/NoGo task in order to take a base measure of the mean Go Reaction Time (Alderson et al., 2008; Cohen et al., 2010). Instructions for the Go/NoGo task were presented orally with a simple form. We used a standardized version of the Go/NoGo task, using green airplanes as go signals. Participants were told to hit the down arrow of the computer keyboard when the go signal appeared in the screen, and avoid responding when seeing a smiley face (the NoGo signal). NoGo's were randomly presented, but not consecutively 25% of the times a stimuli appeared.

Behavioral inhibition was examined using a SST paradigm that requires the cancellation of an already triggered go response. The experiment involves a routine motor reaction (hit a key) to a frequent go stimulus, with occasional cancellation of the routine response after an infrequent stop signal (Logan, 1994).

**Figure 1:**
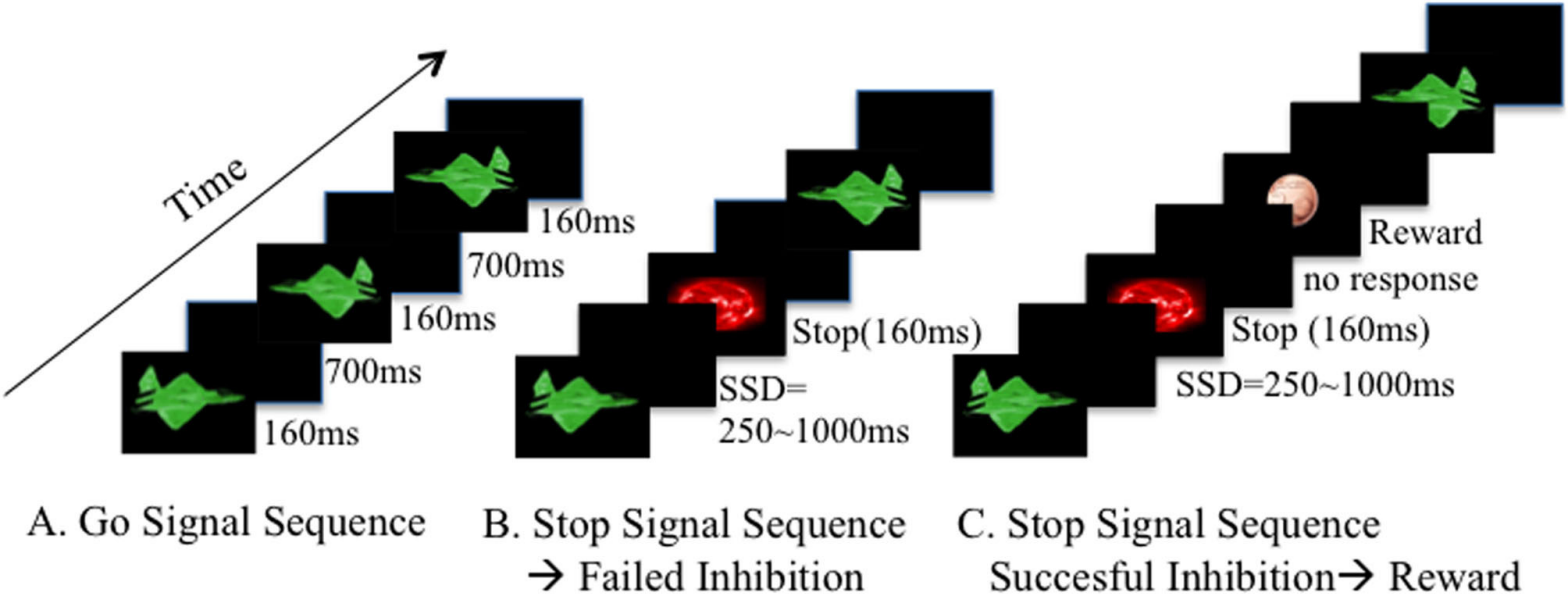
**Experimental paradigm description**. The Stop Signal Task is composed by a Go stimulus, and a Stop signal after some Go stimuli, demanding the cancellation of an already triggered Go response. In our paradigm, each block has an estimated duration time of 8 min, with slight individual variations depending on the participant responses. Two hundred and ninety Go stimuli (green planes ~160 ms) and 59 Stop Signals (red planets ~160 ms) were presented in a counterbalanced order. **(A)** Go signal sequence: participants were asked to hit the right or left arrow of a keyboard, depending on the orientation of the plane. **(B)** Stop Signal appears after the Go Stimulus, in a delay between 250 and 1000 ms. This is called the Stop Signal Delay (SSD). If the participant fails to inhibit, no feedback is shown and the task continues with a new Go stimulus. **(C)** If the participant manages to withhold the motor response after the Stop Signal, a virtual reward is shown. Each block has a specific reward magnitude (smiley, 5 or 50 cts). Order of presentation of each block depends on the type of Study (see Pilot Study, Study One and Study Two for specific descriptions).

Instructions for the SST were presented in a standardized paper form. Participants were told that they were going to perform a video game-like task to determine how fast they were. They were told about the length of the task (6 acquisition blocks for the pilot study, 3 blocks for study one, and 4 blocks for study two) with a short pause between blocks (see Figure 1).

After reading the instructions, participants were asked to repeat the instructions to the evaluator and questions were answered. A brief training block of the SST without feedback was undertaken in order to ensure that the instructions had been fully understood.

In the present study we modified the SST developed by Rubia et al. (2003), which is, in turn, a faster visual variant of the Tracking SST (Logan et al., 1997).

As a modification to the SST, feedback is presented after each successful inhibition. Six types of feedback were exhibited: a smiley for the no-rewarded blocks, 1, 5, or 10 cents coin for the low incentive blocks, and a 20 or 50 cents coin for the high incentive blocks. The type of feedback was constant during each block. Participants were told about the order of presentation of rewards before the execution of the task.

These feedbacks were combined to build particular conditions, conducted along three experiments: pilot study, study one, and study two (refer to each experiment descriptions after the Methods section).

Participants performed successive acquisition blocks, ~8 min each (6 blocks for the pilot study, 3 for the study one, and 4 for the study two). In each block two hundred and ninety green airplanes (Go-signals) were displayed on the middle of the screen for 300 ms. Participants were instructed to respond as quickly as they could by making left and right button presses (according to the direction of the plane). After the airplane, there was a blank screen for 700 ms except at ~20% of times. This accounted for the stop occurrences in the form of 59 red planets. They appeared after the airplane, at variable intervals, which corresponds to the SSD. The SSD changes in 50 ms steps, incrementing after successful inhibition, and decrementing after failed inhibitions. The SSD offset ranged from 250 up to 1000 ms.

Feedback was presented after each successful inhibition, at an offset of 250 ms after the stop stimulus disappearance. Predefined pseudo-randomized ISI occurred at 1600, 1700, 1800, 1900, or 2000 ms intervals and was not varied dynamically to balance for the frequency of successful vs. unsuccessful inhibition (Hampshire et al., 2010). For successful inhibition trials, where the feedback is presented, ISI expands dynamically depending on the predefined pseudo-randomized interval, and going up to 2400 ms till the next go stimulus trial presentation.

### Data analyses

Statistical analyses were performed with SPSS (IBM SPSS Software 19.0 Version, 2010). All data were checked for outliers, normal distribution, and homogeneity of variance. Critical alpha was set at 0.05 but frequently adjusted using Bonferroni corrections.

The dependent variables were three response time measures (MRT, SSD, and SSRT), and four task performance measures (number of failed inhibitions, missed go's, wrong keys, and number of rewards).

All variables were ready to be analyzed after recording, with the exception of the SSRT that was generated through a mathematical model proposed by Logan et al. (1997), following a subtraction of the MRT minus the SSD (formulae SSRT = MRT − SSD) (see Congdon et al., 2012, for a nice and detailed mathematical explanation).

A common model of a mixed ANOVA design was applied to the three studies. Each dependent variable was analyzed through the within-factors “order of blocks” (1, 2, 3 … given by the acquisition block order), “type of reward” (no reward, low reward, high reward) and the between-factor “condition” (increasing reward, decreasing reward).

Given the fact that each group condition was formed by different participants, we considered important to conduct a mixed ANOVA model in order to explore the behavior of the totality of participants, and then, separated One-Way ANOVA models, for each group condition (Increasing or Decreasing Reward) to better grasp the inner modulations of each independent condition, independently of the interaction effects (between groups) explored through the Two-Way ANOVA.

Since most task performance measures did not show a normal distribution, non-parametric tests were conducted for each independent group (Increasing and Decreasing Reward) through Wilcoxon paired-sample tests. Comparisons between each task measures and the condition groups for Increasing vs. Decreasing Reward groups were conducted through Kruskal–Wallis independent-sample tests (and corrected using Bonferroni).

## Materials

Each participant performed the task on a DELL personal computer equipped with an Intell 2 processor. Individuals were seated 1 m from a 20″ screen, the nose aligned with the fixation cross so the reward stimuli would fall in the center of the visual field.

The stimuli presentation was programmed in Visual Basic 6.0. Each stimulus was presented against a black background at the center of a 15 inches standard screen.

The test was performed in a testing room, artificially lighted. At the beginning of the task, the participants underwent a short practice block, ensuring the correct visualization of every stimulus; luminosity was kept constant in the stimuli with no ambiguity. There was no need to measure the luminosity screen.

### GO/NOGO

Seventy-seven participants (*n* = 38 for study one, *n* = 39 for study two) underwent the Go/NoGo version in a single acquisition block of 8 min of duration. The Go/NoGo Task was not applied to the pilot study participants.

Mean and standard deviation to reaction time scores of the Go/NoGo Task per individual served as normalization parameter to the reaction time's obtained through the SST. Mean values were very consistent (study one *MRT* = 282 ms, *SD* ± 72; study two *MRT* = 223 ms, *SD* ± 41).

### The pilot study

The aim of the pilot study was to test the effect of several rewards on inhibitory control as measured by different monetary rewards during the SST.

#### Procedure (pilot study)

Twenty one participants [mean age 31 (*SD* = 5.2), gender ratio was 1.1] participated from the pilot study. Our in-house version of the SST was applied in 6 blocks. Four types of reward feedback were introduced: a no monetary reward with smiley, 1, 10, and 20 cents coins. Smiley feedback was always presented at the beginning of the protocol and for the odd blocks (blocks 1, 3, and 5). Monetary feedback was given on the even blocks (blocks 2, 4, and 6). The reward magnitudes for these even blocks were assigned in a random manner.

Given the distribution of reward through the task, participants were categorized in 3 conditions, relying on the progression trends of the reward magnitudes in time: “increasing condition” when the participant received low rewards at the beginning and then increasing reward magnitudes, “decreasing condition” when going from high rewards to low rewarded blocks, and “variable condition,” with no specific reward progression pattern.

#### Analysis (pilot study)

Two mixed ANOVA models were applied with the aim of explore diverse aspects of the reward effect over the inhibition profile. As described below, we examined the inhibition profile by response time and performance measures (MRT, SSD, SSRT, number of failed inhibitions, missed go's, wrong keys, and rewards, as described in the general methods section).

First, a One-Way ANOVA model was conducted to look at the influence of the reward magnitude *per se* on the time measures of the task, regardless of the order of reward type assignment or group type. Second, a 4(no reward, 1, 10, 20 cts) by 3(increasing condition, decreasing condition, variable condition) ANOVA was conducted to analyse the influence of the time history of reward assignment over the inhibition dependent variables.

#### Results (pilot study)

The One-Way ANOVA revealed no modulatory effect of reward on the time measures of the SST although the descriptive results suggested differences that prompted further analyses.

The Two-Way mixed ANOVA did not show a significant effect of group for MRT or SSD. Even though there was a trend for group effect in SSRT [*F*_(5, 20)_1, 73, *p* = 0.052]. There was no interaction effect between the group and the reward assignment conditions. However, this first experiment was not set to explore the group difference and for this was underpowered. We further test the hypotheses of group type and order of reward on the subsequent experiments.

Post paired tests for time measurements corrected for multiple comparisons, (MRT, SSD, SSRT) suggested differences between the first two blocks and the rest of the acquisition blocks set. The contrasts were significant for the SSD between first and second block [*df*_(1, 20)_, *p* = 0.002] and the SSRT between the first and the last block [*df*_(1, 20)_, *p* = 0.001]. Task performance variables showed a consistent and progressive improvement in gains, accuracy and less error.

#### Pilot study conclusions

Pilot study analysis primarily showed a ceiling performance profile in all of the dependent variables, supposedly not influenced by the different reward magnitudes.

The progressive improvement trend most probably suggests a learning effect acquired through the lengthy task. This improvement seems to occur at a critical point where most of the performances had no more room for improvement. The SSRT appeared to improve till the last acquisition block. This training effect over the SSRT is not in agreement with previous studies theorizing that this value is a stable inhibition landmark (Cohen et al., 2010).

## Study one: effect of reward magnitude and reward history

The aim of study one was to determine whether there is a modulatory effect over performance induced by different reward magnitudes, and the extent to which the order of presentation of rewards may modulate performance on subsequent blocks.

### Procedure (study one)

Thirty-eight participants [mean age 24 (*SD* = 4), gender ratio 1.1] were included. The study design was programmed after a close analysis of the pilot results. The fallout was a briefer protocol, a clearer reward assignment with only two types of monetary feedback sequences, instead of the four applied on the pilot study. The random reward assignment was replaced for a clear design where participant where allocated to an Increasing or Decreasing Reward Group.

The outcome was a three-block protocol with the modified version of the SST. Participants were distributed in two groups corresponding to either Increasing or Decreasing Reward. Participants in the increasing reward condition began with a no monetary reward (smiley face feedback) block, second block, monetary feedback was 5 cents, and third was 50 cents. Participants in the Decreasing Reward condition undertook the same number of acquisition blocks, but rewards were presented in the reverse order (first block showing a 50 cents feedback, second block 5 cents, and finally the smiley). All participants were aware of the reward presentation order before beginning the task.

### Results (study one)

#### Time performance measures

We first performed a One-Way ANOVA to evaluate the influence of the reward magnitude *per se* over the task measures regardless of the order of reward or group. The ANOVAs for SSD, MRT, and SSRT showed no significant differences between rewards, similarly to the initial results from the pilot study.

Two-Way mixed ANOVAs were conducted for each time measures (MRT, SSD, SSRT). A 2^*^3 design was applied, given the two condition groups (increasing and decreasing rewards), the three blocks of reward levels (first, second, and third block in one of the two orders defined by the design), or the three reward magnitudes (smiley, 5 cts, 50 cents) per Group. The first permitted the assessment of the effect of Order of reward and the second to test the reward effects *per se* while taking into account the Group factor. Means and SD are reported on Table 1.

**Table 1:** **Study One: Increasing and decreasing conditions**.

**Order**	**Increasing condition (*n* = 18)**			**Decreasing condition (*n* = 20)**		
	**1**	**2**	**3**	**1**	**2**	**3**
**Reward size**	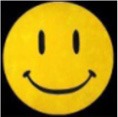	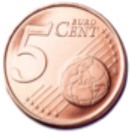	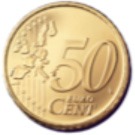	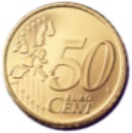	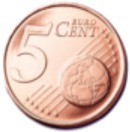	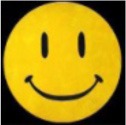
	***M* ± *SD***	***M* ± *SD***	***M* ± *SD***	***M* ± *SD***	***M* ± *SD***	***M* ± *SD***
MRT (ms)	969 ± 224	1056 ± 261	1005 ± 263	1036 ± 216	1073 ± 228	1061 ± 208
SSD (ms)	700 ± 210	823 ± 197	835 ± 156	714 ± 229	778 ± 228	808 ± 221
SSRT (ms)	269 ± 106	232 ± 190	170 ± 210	321 ± 93	294 ± 133	253 ± 147
Failed stops (*n*°)	23.2 ± 6.2	21.9 ± 8.0	18.6 ± 8.5	24.6 ± 5.7	20.7 ± 7.7	18.8 ± 8.4
Missed go (*n*°)	7.4 ± 6.5	6.9 ± 5.4	8.8 ± 7.3	7.1 ± 8.3	7.2 ± 4.7	5.1 ± 3.3
Wrong keys (*n*°)	3.3 ± 3.9	1.9 ± 2	3.5 ± 3.4	1.5 ± 1.8	2.1 ± 1.9	1.3 ± 1.3
Rewards (*n*°)	30.7 ± 5.9	32.7 ± 7.2	36.3 ± 7.8	32 ± 5.4	33.5 ± 6.4	36.7 ± 8.4

*All Measures of performance in speed and accuracy*.

*MRT, Mean reaction time; SSD, Stop signal delay; SSRT, Stop signal reaction time*.

**Figure 2:**
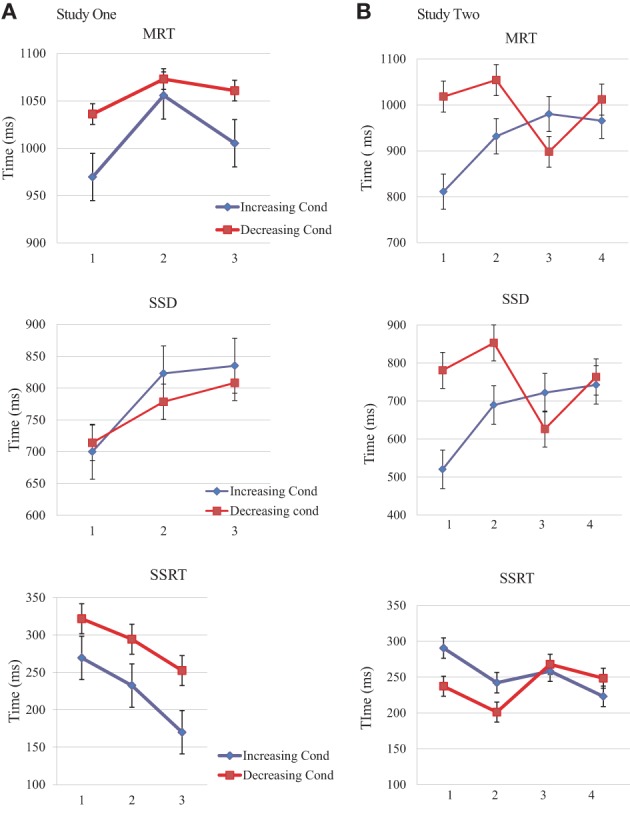
**Order effects of Reward for MRT, SSD, and SSRT**. MRT (top), SSD (middle), and SSRT (bottom) means and standard errors for Study One and Study Two. **(A)** Study One blocks in the order of presentation (1, 2, and 3). Increasing condition participants (blue lines) received a smiley for successful inhibition on the first block, then 5 cents and finally 50 cents for the last block. Inverse reward for the decreasing condition group (red line). **(B)** Study Two blocks in the order of presentation (1, 2, 3, and 4). Increasing condition participants (blue lines) received two 5 cts block for successful inhibition and then two blocks of 50 cents. Inverse reward for the decreasing condition group (red line).

Standard deviation scores seemed different for the time measures over the two condition groups, suggesting higher variance among participants in the Increasing reward condition (see Table 1). Despite similar slope changes for both condition groups, performance appeared slower and less variable for the Decreasing condition group (albeit not significant), we suspected from a different strategy for each condition group (Figure 2A).

The ANOVA model for the Reward magnitude analysis (independent of Order of reward), revealed a clear Interaction effect for SSD [*F*_(2, 72)_ = 18.21, *p* < 0.001] and SSRT [*F*_(2, 72)_ = 7.52, *p* = 0.001] between Reward magnitude and Group. There was no effect of reward *per se*.

The ANOVA testing Group, Order of reward and interaction showed a robust effect of Group for all three time measures (MRT, SSD, and SSRT) but no significant main effect of the Order of reward. Likewise, no interaction effect between the Group time measures and Order of reward was revealed (See Table 2).

**Table 2:** **Study One: Two-Way ANOVA for time performance measures**.

	**Main effect of group**	**Main effect of order**	Group*Order interaction
	**(*df* = 1.37)**	**(*df* = 1.37)**	**(*df* = 2.72)**
	***F(p)***	***F(p)***	***F(p)***
MRT (ms)	6.62 (0.002)	0.47 (0.49)	1.19 (0.307)
SSD (ms)	18.5 (<0.001)	0.006 (0.94)	1.49 (0.23)
SSRT (ms)	7.87 (0.001)	1.64 (0.207)	0.177 (0.83)

*MRT, Mean reaction time; SSD, Stop signal delay; SSRT, Stop signal reaction time*.

*Order refers to the reward order assignment for each condition group*.

*Post-hoc* comparisons (Bonferroni corrected) were performed between the three blocks independently from the factor “Group,” assessing the effect of order *per se*, showing that paired comparisons between MRTs on blocks 1 and 2 significantly differ [*df*_(1, 37)_, *p* < 0.001], but not MRT on block 1 and 3 [*df*_(1, 37)_, *p* = 0.36] or block 2 and 3 [*df*_(1, 37)_, *p* = 0.19]. Same paired-tests for SSD revealed significant differences between blocks 1 and 2 and block 1 and 3 [*df*_(1, 37)_, *p* < 0.001] but not between blocks 2 and 3 [*df*_(1, 37)_, *p* = 0.031]. Along with the ANOVA, these results suggest an effect specifically due to the chronological progression of the task, independently of the reward magnitude assigned on each block.

SSRT paired comparisons did not significantly differ between the first two blocks: block 1 vs. block 2 [*df*_(1, 37)_, *p* = 0.70], but they significantly differed between the first and last blocks block 1 vs. block 3 [*df*_(1, 37)_, *p* = 0.003], and showed a trend between the second and third blocks [*df*_(1, 37)_, *p* = 0.017].

Additional *post-hoc* comparisons (Bonferroni corrected) were also performed between the three blocks from the factor “Reward,” assessing the effect of reward magnitude *per se*, revealing a lack of significant effects for MRT, SSD, or SSRT measurements.

To further explore the effect of the reward magnitudes and order but independently for each Group, One-Way ANOVAs were conducted for Increasing and Decreasing rewards, separately. No significant differences were observed for time measures (MRT, SSD, and SSRT). So we cannot conclude that reward magnitude modulates inhibitory control when probing each group alone.

To disentangle the effects of the blocks we tested each pair of blocks between the two condition groups inside the ANOVA model with an univariate analysis for paired comparisons (Bonferroni corrected, block 1 from Increasing Reward group and Block 1 from Decreasing Reward group, and the same for blocks 2 and 3). No significant differences were revealed when comparing same presentation order blocks between the two condition groups. No firm conclusion can be drawn from the differences between the Decreasing and Increasing groups in pair comparisons either.

#### Task performance measures

Errors were indexed by counting the number of inhibition errors (failed stops), missed go signals (missed go), and precision errors for the left-right decision (wrong keys). Gains correspond to the number of rewards.

Performance measures did not exhibit a normal distribution. Non-parametric tests (Kruskal–Wallis for independent samples) comparing each performance measure between the two condition groups did not show any significant differences.

Wilcoxon test for related samples comparing performances between blocks for each group separately, showed significant differences for the inhibition errors (number of failed stops) between the first and last blocks [*df*_(1,37)_, *p* = 0.006] and between the second and last block [*df*_(1, 37)_, *p* = 0.004] for the increasing condition. This may correspond to a training effect as well as a motivational effect with the increasing reward magnitude. No significant differences were detected for the Missed Go or Wrong Key scores for the paired comparisons in the increasing condition.

Decreasing condition performance for the number of failed inhibitions were significantly different for paired blocks 1 and 2 [*df*_(1, 37)_, *p* = 0.003] and blocks 1 and 3 [*df*_(1, 37)_, *p* = 0.001], as well as for the number of Rewards between the first and last blocks [*df*_(1, 37)_, *p* = 0.007]. A progressive improvement of inhibition errors and reward raw scores was observed.

Missed Go's paired comparisons were significantly different for the last two blocks inside the decreasing condition [*df*_(1, 37)_, *p* = 0.007]. Missed Go's raw scores also show an improvement through the task, as for the number of failed inhibitions and rewards.

Again, against the hypothesis, participants from the decreasing condition group did not decrease their performance with diminishing rewards. We favor the explanation of the training/learning effect.

### Comments on study one

The results suggest a modest modulatory effect of reward on performance for both condition groups, with a strong effect from the start when the highest reward is received in the first block of the task. A learning (or practice/training) effect may be the cause for the improvement of performance independently from rewards and its order.

Despite the clear main effect of group, the order of reward assignment (or an interaction effect between the groups) was not demonstrated. The main effect of group cannot necessarily be attributed to the fact that participants were exposed to one or the other reward assignment condition. Notwithstanding similar slope changes for both condition groups, averages seemed slower and less variable for the decreasing condition group (albeit not significant), leading us to suspect that each group may be applying a different strategy from the first block on.

Decreasing condition participants appeared to exhibit better global performances from the first block and consequently seemed more resistant to reward modulation afterwards. This early effect, which we refer to as the “kick start effect,” may be caused by the high reward received in the first block. That is, the reward in the first block defines the primary performance, probably creating a mental set, and shaping a strategy that favors withholding of responses, and remains resistant to reward changes afterwards. The general performance seemed also modulated by a learning effect that was evident in the pilot study, where increasingly better scores were reported, regardless of reward or order of presentation of rewards. Participants may have learnt to proactively withhold their responses. The training effect trend was suggested by the SSD, number of failed inhibitions, and reward values for both condition groups, given by an apparently progressive improvement through the three blocks.

MRT for the increasing condition group, showed a steep slope between the first and the second block, corresponding to the transition between a no-rewarded condition to the first monetary reward condition. It could be hypothesized that the motivational boost effect of the first reward is strong. Participants in the increasing condition group also showed a clear slowing down of progression in the task (SSD), from block to block maybe signaling a better inhibitory capacity. However, it may be due to the training effect, which is also observed in performance measurements of the number of failed inhibitions. The suggested training effect could be masking the reward magnitude effect for the increasing condition group. SSRT scores also exhibited an improvement trend as suggested by the significant differences between first and second, and first and third blocks, also evidenced for both reward conditions, and the training effect could be at the origin of this trend or be the main contributor.

SSRT scores are the hallmark of the inhibition process measured through the SST, participants on the increasing condition group progressively obtained better inhibition scores (269 ms for the first trial, up to 170 ms for the last). The enhancement of the inhibition capacity has been described between reward cues and no-reward cues in the SST (Scheres et al., 2001; Lijffijt et al., 2004; Nigg, 2005). It may be difficult to disentangle whether the SSRT improvement on this experiment was induced primarily by the presence of increasing rewards or by the training effect.

Decreasing condition participants appeared to exhibit better global performances from the first block and consequently seemed to change to a lesser degree with subsequent blocks. This effect of high reward at the beginning of the task seemed to modulate performances from then on, reflecting our proposed “kick start effect.”

Time measurements were not significantly different from block to block inside the same condition group. Even though MRTs had a slower starting point compared to increasing condition group (Figure 2). Likewise, SSRT were slower from the beginning, in comparison with SSRT from the increasing condition group (Figure 2).

In sum, the effect of the reward magnitude is suggested by an improvement in the global scores for the increasing reward group, with best performances for the higher rewarding blocks. Nevertheless, this magnitude reward effect was not backed up by an interaction effect, suggesting that there are other major factors influencing reward processing and inhibition. A training effect is a more likely explanation given a shared improvement pattern for Increasing as well as for Decreasing condition group. Moreover, a quick modulation of performances from the starting block, suggests a particular modulatory effect given by the impact of the history of reward assignment. In support of this “kick start” interpretation, it was observed that the Decreasing condition seemed to be influenced from the high-reward first block, thereby increasing the baseline performance.

## Study two: effect of reward magnitude

The aim of Study Two was to determine the effect of different levels of reward magnitudes (low vs. high rewards) and whether those contrasting magnitudes produced a modulation dependent on individual differences in SSRT performances.

### Procedure (study two)

Thirty-nine healthy participants underwent a two-rewards protocol [mean age 24.7(*SD* = 4.5), gender ratio 1:1] presented in a four-block task: increasing reward (*n* = 20) and decreasing reward (*n* = 19) contingencies.

A four-block protocol was designed to contrast high and low rewards depending on order of presentation. Participants were allocated to one of two groups corresponding to either Increasing or Decreasing Rewards. In this experiment, the Increasing Group had four blocks with reward order as follows: 5, 5, 50, and 50 cts; while the Decreasing Group order was: 50, 50, 5, and 5 cts (see Table 3, for experimental design, means and STDs). There were no no-reward blocks in this second experiment and we also controlled for learning effects by including a repetition in each reward condition. All participants were aware of the reward presentation order before beginning the task.

**Table 3:** **Study Two: Increasing and decreasing conditions**.

**Order**	**Increasing condition (*n* = 20)**				**Decreasing condition (*n* = 19)**			
	**1**	**2**	**3**	**4**	**1**	**2**	**3**	**4**
**Reward size**	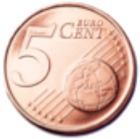	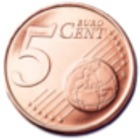	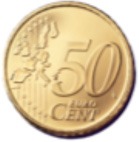	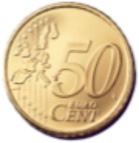	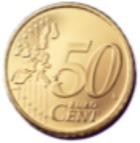	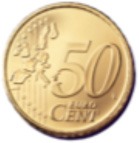	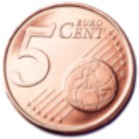	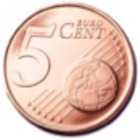
	***M* ± *SD***	***M* ± *SD***	***M* ± *SD***	***M* ± *SD***	***M* ± *SD***	***M* ± *SD***	***M* ± *SD***	***M* ± *SD***
MRT (ms)	811 ± 263	932 ± 310	980 ± 315	965 ± 291	1018 ± 256	1054 ± 211	898 ± 241	1012 ± 255
SSD (ms)	520 ± 300	689 ± 299	722 ± 285	742 ± 265	781 ± 233	853 ± 166	626 ± 276	763 ± 238
SSRT (ms)	291 ± 156	242 ± 203	258 ± 162	223 ± 187	237 ± 168	201 ± 168	268 ± 146	248 ± 142
Failed stops (*n*°)	23.0 ± 7.8	17.4 ± 7.6	15.3 ± 6.5	15.1 ± 6.8	15.3 ± 7.2	13.9 ± 5.9	20.0 ± 7.1	15.6 ± 7.2
Missed go (*n*°)	5.6 ± 3.8	6.1 ± 4.9	5.3 ± 4.4	5.3 ± 3.6	6.1 ± 5.2	6.4 ± 7.0	4.0 ± 2.3	4.6 ± 3.2
Wrong keys (*n*°)	3.8 ± 4.5	2.8 ± 3.0	2.2 ± 2.8	2.8 ± 3.1	2.4 ± 2.5	2.4 ± 3.8	4.3 ± 5.6	2.7.0 ± 3.0
Rewards (*n*°)	28.0 ± 6.7	33.3 ± 8.2	36.4 ± 9.2	35.4 ± 8.5	35.9 ± 8.2	37.6 ± 8.8	32.0 ± 7.6	36.8 ± 8.3

*All Measures of performance in speed and accuracy*.

*MRT, Mean reaction time; SSD, Stop signal delay; SSRT, Stop signal reaction time*.

### Results (study two)

#### Time performance measures

We first performed a One-Way ANOVA to evaluate the influence of the reward magnitude *per se* over the task measures regardless of the order of reward or group. The ANOVAs for SSD, MRT, and SSRT showed no significant differences between rewards, confirming the initial results from the pilot study and Study One.

Two-Way mixed ANOVAs were conducted for each time measures (MRT, SSD, SSRT), the first aiming and testing the reward effect *per se* by group and the second to evaluate the order effects. The Reward 2^*^2 mixed-model ANOVA was conducted between the 2 conditions (increasing and decreasing reward) and the 2 reward magnitudes (5 and 50 cents) and showed a main effect of group for MRT [*F*_(1, 37)_ = 18.75, *p* < 0.001] and SSD [*F*_(1, 37)_ = 29.4, *p* > 0.001], but no effect of Reward, and no interaction effects.

The 2^*^4 mixed-model ANOVA was applied to test the Order effects, with 2 conditions (increasing and decreasing Reward) and 4 blocks per time measure (MRT, SSD, SSRT). There were significant main effects of group in the all the time measures (MRT, SSD, SSRT), and significant interaction between group and order of reward assignment for MRT and SSD but not for SSRT (see Table 4).

**Table 4:** **Study Two: Two-Way ANOVA for time performance measures**.

	**Main effect of group**	**Main effect of order**	Group* Order interaction
	**(*df* = 1.38)**	**(*df* = 1.38)**	**(*df* = 3.111)**
	***F(p)***	***F(p)***	***F(p)***
MRT (ms)	5.81 (0.001)	0.79 (0.38)	14.94 (<0.001)
SSD (ms)	4.45 (0.042)	1.29 (0.26)	24.17 (<0.001)
SSRT (ms)	1.53 (0.020)	0.11 (0.74)	1.28 (0.284)

*MRT, Mean reaction time; SSD, Stop signal delay; SSRT, Stop signal reaction time*.

*Order refers to the reward order assignment for each condition group*.

Pairwise comparisons (Bonferroni corrected) *post-hoc* tests between the four blocks for the combined group (grouping the two conditions) tested the training effects *per se*, revealing significant differences for MRTs between blocks 1 and 2 [*df*_(1, 38)_, *p* = 0.003], blocks 1 and 4 [*df*_(1, 38)_, *p* = 0.007] and blocks 3 and 4 [*df*_(1, 38)_, *p* = 0.008]. SSDs post-tests were also significant for the paired comparisons between blocks 1 and 2 [*df*_(1, 38)_, *p* < 0.001], blocks 1 and 4 [*df*_(1, 38)_, *p* = 0.008], and the last two blocks, block 3 vs. block 4 [*df*_(1, 38)_, *p* = 0.005]. Pairwise comparison for the SSRT scores did not significantly differ. This exploratory analysis showed small but significant effects between blocks despite a negative main effect of order in the ANOVA. These findings also suggest dissimilar strategies between the two condition groups, supported by the interaction effect of group and order of presentation of rewards.

To further explore the effect of the reward magnitudes per condition group, One-Way ANOVAs were conducted for each independent condition group (Increasing or Decreasing Reward). No significant differences were observed for any time measures (MRT, SSD, and SSRT).

To better define the possible origin of the differences in the Order ANOVA, we conducted univariate comparisons in the ANOVA model (Tukey HSD) for first blocks, second, third and fourth blocks between the two conditions (block 1 from Increasing reward group vs. block 1 from Decreasing reward group, repeated for each of the 4 blocks). No significant differences were observed between each time-related block between the two conditions, for the MRT or the SSRT. However, univariate paired comparisons between time-related blocks on both condition groups, revealed that SSDs significantly differed between the first two blocks [*df*_(1, 38)_, *p* = 0.004], suggesting a different strategy at the beginning of the task. This result may add some support to the kick start effect found in Study One.

While MRT and SSD showed significant interaction effects, SSRT failed to show differences. Furthermore, a lack of differences between blocks was probed independently through the One-Way ANOVA analyses.

An interpretation of these results will be presented in the discussion part, however, we think these findings suggest that the SSRT, as a compound measure, may lack sensitivity, and breakdown on MRT and SSD bears the potential to provide useful information on the behavioral adjustments, otherwise hidden by the SSRT scores.

#### Task performance measures

Performance measures (Number of Failed Stops, Missing Go's, Wrong Keys, and Number of Rewards) did not exhibit a normal distribution. Non-parametric tests (Kruskal–Wallis for independent samples) showed significant differences between the number of failed stops for the first block between the two groups [*df*_(1, 38)_, *p* = 0.006], as well as for rewards also for the first block comparison [*df*_(1, 38)_, *p* = 0.004], consistent with the pairwise comparison between SSD time measurements. These results—again—support an early effect of the reward (kick start effect).

Wilcoxon test for related samples (Bonferroni corrected for multiple comparisons) were conducted to compare performances within each condition group. Failed Stops and Rewards were significantly different in several pair comparisons among blocks in the increasing condition reward group [failed stops between blocks 1–3 [*df*_(1, 38)_, *p* = 0.001], blocks 1–4 [*df*_(1, 38)_, *p* = 0.003]; number of rewards between blocks 1–2 [*df*_(1, 38)_, *p* = 0.002], blocks 1–3 [*df*_(1, 38)_, *p* = 0.001], Blocks 1–4 [*df*_(1, 38)_, *p* = 0.001], blocks 2–3 [*df*_(1, 38)_, *p* = 0.004]. Missed Go and Wrong Keys did not differ in any comparison. For the decreasing reward group, robust differences were found between the second and third blocks for Failed Stops [*df*_(1, 38)_, *p* = 0.002], Wrong Keys [*df*_(1, 38)_, *p* = 0.006], and Rewards [*df*_(1, 38)_, *p* = 0.009]. These findings support the hypothesis of the modulation of inhibitory control with reward magnitude since significant differences were found primarily among the strongest rewarded blocks (blocks 1 and 2) and the drop of reward magnitude in the third and fourth block.

### Comments on study two

Analysis of Study Two revealed a robust main effect of group like in Study One, supporting the hypothesis of modulation of inhibitory control by history and context of reward, but also showed an interaction effect between group and order of presentation of rewards. In this experiment, the reward assignment seems to have induced differences in how performances change. However, the order effect *per se* was not significant.

SSD comparisons for the same block between groups showed a marked difference in Blocks 1 and 2; MRTs are clearly slower for the first blocks on the decreasing condition, suggesting an immediate behavior modulation for the highest rewarded blocks. When observing MRTs for the higher reward blocks on the increasing condition groups, there was also a withholding pattern, but the scores were not as slow compared to those obtained for the same rewarded blocks on the decreasing condition. These observations again support a kick-start effect induced by the highest reward at the beginning of the task (Figure 2B).

**Figure 3:**
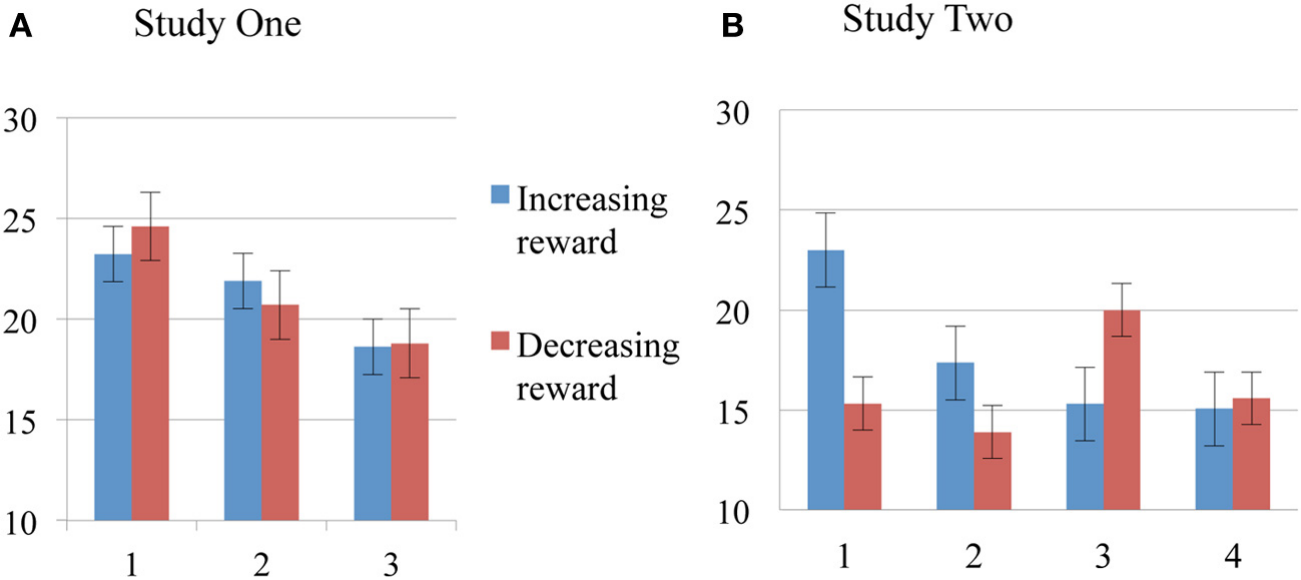
**Order effects of Reward for Failed Inhibitions**. Means and standard errors for Study One **(A)** and Study Two **(B)**. Blue bars for Increasing Groups, red bars for Decreasing Groups.

Performance measures suggest the modulatory effect of reward magnitude history highlighting two main phenomena: (1) participants in the Increasing condition improved their performances in a progressive manner throughout the task, and (2) participants in the Decreasing condition had a good performance from the beginning of the task (high reward) and a dramatic fall in all measures when they transitioned from high to low reward blocks. Furthermore, Decreasing group scores were higher, not only from the beginning, but also when compared to high reward blocks from the Increasing Group. This finding suggests a similar kick-start effect in both studies Two and One (Figure 3).

## Discussion

The aim of this study was to investigate the behavioral effect of reward contingencies in the SST by manipulating the magnitude and order of reward. Little research has been done manipulating different reward magnitudes in an inhibition task (but see Shanahan et al., 2008) despite the common use of punishment and reward in learning to stop a particular behavior or inhibit an urge (Ridderinkhof et al., 2004). We argue that the experimental design from the two studies presented here provide deeper insight into the motivational mechanisms of the inhibitory processes, pushing experimental contingencies beyond the primary executive-motor dimension and shedding light into the mechanism underlying the modulation of cognitive control. Our study introduced multi-level reward magnitudes along with a dynamical presentation of those reward contingencies over two different experimental conditions. We intended to obtain additional clues to understand the motivational aspects of the manipulation of reward magnitudes in the same inhibition task. What is the impact of the reward magnitudes on cognitive inhibition? What is the overall motivational effect of giving rewards, independently of their magnitudes? Does reward size matters? We predicted two effects: a modulation of inhibition through reward *per se* and the modulation of the history (through order of presentation) of those rewards. The results observed for study one provided weak evidence in support of our hypothesis that there is a modulation on behavioral inhibition depending on the reward magnitude and order. Study two provided a more consistent confirmation of our reward effects hypotheses. Both studies demonstrated a strong modulation effect of the history of reward assignment.

In the pilot study, reward contingencies were masked by the randomization of reward magnitude blocks and furthermore, the experimental design was long (six blocks), allowing the development of a learning pattern that was evident across most of the performance measures, and particularly for the SSRT. Whilst the inhibitory measures seemed mostly independent of reward contingencies, the fact that they varied with practice, in conjunction with the results of other two experiments, demonstrate that the SSRT is not as stable a trait as originally claimed. Scheres et al. (2001) showed evidence on the improvement of SSRT scores due to reward contingencies on ADHD children. However, the SSRT seemed weak and unstable in the pilot study, leading us to postulate that by exploring the SSRT building blocks, the MRT and SSD, there is potential to better understand the inhibition modulation by reward.

The original hypotheses proposed a change, proportional to the reward magnitude and independent of the presentation order, nonetheless, it quickly became evident after the pilot study exploration that more specific hypotheses regarding the effects of reward on inhibitory control were needed to account for the results obtained and expected. This first pilot analysis permitted the design of specific experiments to test the effect of history of reward, and the reward magnitude itself.

Study One provided little evidence for the impact of reward magnitudes but strongly suggested a kick-start effect, a result that accords with the pilot study. A learning effect was also evident in Study One, with a progressive improvement on raw scores for both groups, that is, independent of the reward size assignment. Study Two offered clearer evidence of the effect of the reward modulation. We believe this discrepancy to be explained by the design of study being more prone to the masking effects of improvements due to task learning.

Studies One and Two both showed that participants on the decreasing conditions, that is, who start with higher rewards, presented better global scores on all measures. This result was unexpected and based on this we have proposed that there was an early modulatory effect induced by the reward. In study 1, the early modulatory effect was sustained across blocks even if rewards decreased or disappeared. Thus, we termed it the “kick start effect,” as it seems to have a lasting influence over performances throughout the task. The results from study's one and two suggest that this kick-start effect works via a reward “boost,” which impacts the behavioral markers of inhibition of the executive process in play during the SST. Thus, the presence of reward at the beginning of the task can lead to higher cognitive control over performance by moving the threshold of the capacity to withhold a response.

The results of study two demonstrated that the reward magnitude modulation seems to be independent of learning or practice effects but highly dependent on context. Participants were able to improve their scores when confronted to a higher reward on the Increasing condition, as expected, but there was a performance decrement from the second to the third block in the decreasing group, not caused by a lack of training but possibly due to a disengagement of motivation: participants that were exposed to 50 cents feedback for each successful inhibition, suffered a fall of 45 cts per trial after the third block. This change of reward seems to induce an override of the motivational effect that cannot be explained by practice.

The results in study one and two clearly suggest that behavioral adjustments may not only be related to the order of the reward magnitude, but also due to a “kick start effect” that modulates performance from the beginning and has consequences throughout the rest of the task. Other authors have described similar ideas in the literature of Stop Tasks with reward contingencies, using other terms such as the “Arousal Effect” (Shanahan et al., 2008) or the “Novelty effect” (Ronga et al., 2013). The concepts are not equivalent since the effects where not alike. Further theoretical efforts, based on wider meta-analyses and new experimental findings should help cement these concepts.

In Study One we were able to induce a modulatory effect of the order of reward magnitude that appeared stronger in Study Two. This effect, or rather its interaction, could have been diminished on Study One by several factors: (a) the presence of no-reward blocks in the same task, (b) a masking effect of the history of previous rewards, induced by the kick start effect observed on the decreasing condition, (c) the learning effect and its interaction. It is difficult to disentangle these probable causes but future experiments will necessarily do so when taking into account these factors in their experimental design.

The SSRT is the major index of inhibition pattern obtained for the SST. Many studies utilizing the SST have the tendency to report primarily the SSRT values as noted on the meta-analysis by Alderson et al. (2008). It is important to note that the SSRT is a combined measure obtained indirectly by the calculation of the optimal time up to which inhibition is still possible, according to a given SSD. Comparative analysis using only SSRT values for groups under different conditions may leave out the dynamical changes observed over MRT and SSD. The underlying idea of the SSRT is that it combines the reaction times and the recent history of the response withholding in one compound measure, however, in the present studies, we found no significant SSRT differences that created the impression of an absence of modulatory effects of reward contingencies. However, a more complete analysis revealed hidden patterns behind the MRT, SSD, and errors. We propose that a more detailed inspection of the measures obtained in the SST provides additional information of the differences in inhibitory performance between groups, otherwise hidden by the SSRT raw scores or by limited understanding of task measures.

Electrophysiological and neuroimaging studies should help to explore the underlying mechanisms of inhibitory control modulated by reward (Overbeek et al., 2005; Wiersema et al., 2005). The neurodynamics revealed by evoked potentials may be particularly helpful (Gehring et al., 1990) to determine whether there is a “novelty” or a “saliency” phenomenon with reward, and if there is an ERP magnitude correlation. These questions have been put forward in previous studies on ERN/Ne magnitudes using reward cues (Liotti et al., 2005; Holroyd et al., 2009), some studies even suggest that the ERN/Ne amplitude can also reflect the motivational value of a task, being elicited by individual sensitivities to reward magnitudes, punishments (Boksem et al., 2006; van Meel et al., 2011) or predicted rewards (Yasuda et al., 2004). Methodological adjustments should be taken into account in order to test the order of reward magnitude effect and the kick start effect.

Furthermore, a clinical application of the present protocols may be instrumental in exploring the cognitive (and neurophysiological) signatures in some psychiatric conditions, specifically when impulsivity is one of the core symptoms. There is a growing body of evidence suggesting that reward modulatory effects on inhibitory control could be stronger on Attention Deficit Hyperactive Disorder (ADHD) patients than in the normal population. Our experimental design will be further applied to address this question in ADHD patients in the near future.

### Conflict of interest statement

The authors declare that the research was conducted in the absence of any commercial or financial relationships that could be construed as a potential conflict of interest.
